# Clinician and patient readiness to engage with community health workers at epilepsy care centers

**DOI:** 10.3389/fneur.2025.1580655

**Published:** 2025-04-15

**Authors:** Felicia C. Chu, Barbara C. Jobst, Anna Murray, Trina Dawson, Christine F. Frisard, Barbara Glidden, Wren M. Kaden, Elaine T. Kiriakopoulos

**Affiliations:** 1 Department of Neurology, University of Massachusetts Chan Medical School, Worcester, MA, United States; 2 Department of Neurology, Geisel School of Medicine, Dartmouth College, Hanover, NH, United States; 3 HOBSCOTCH Institute, Dartmouth Health, Lebanon, NH, United States

**Keywords:** epilepsy, social determinants of health, community health workers, health equity, care coordination

## Abstract

**Introduction:**

For patients with epilepsy, medical and social support care gaps may potentially be addressed by clinic-based community health workers (CHWs), particularly for patients most susceptible to health disparities. However, little is known of health professionals’ readiness to integrate CHWs into epilepsy health care delivery.

**Methods:**

An online digital survey was developed and distributed to physicians, nurses and social workers working in 12 comprehensive epilepsy centers in the New England region. Questions in the survey pertained to respondents’ perceptions of working with and addressing clinic needs via a CHW at an epilepsy center. Demographic data were also collected.

**Results:**

Survey results across physicians, nurses and social workers (*n* = 65) revealed low knowledge and experience with CHWs. Epilepsy clinicians are unaware of the scientific evidence showing positive effects of CHW health outcomes. Knowledge of CHW recruitment, training and supervision is low. Despite this, the data collected demonstrate that social determinants of health care gaps exist for epilepsy patients receiving care at epilepsy centers. These gaps could potentially be addressed by a nontraditional healthcare professional, such as a CHW, instead of a social worker or nurse, thus alleviating burden from advanced practice provider members to address other clinically based patient needs.

**Conclusion:**

Despite limited understanding of CHW roles or firsthand experience with CHWs, clinician and patient readiness for integration of CHW was high, with a strong indication that clinicians would refer patients to a CHW and that patients felt the potential for health benefit if provided with assistance from a CHW.

## 1 Introduction

Each individual impacted by epilepsy presents a complex set of medical and psychosocial issues for medical providers to manage in the outpatient clinic setting (1–7). Clinicians often must focus on the medical management of seizures, which does not consistently leave time to address social determinants of health (SDOH), debilitating comorbidities, or the known desire of patients to feel informed about their condition (8, 9). Despite a wealth of evidence indicating that as much as 80% of a person’s health is determined by factors that exist outside of the medical system, our current treatment model does little to address these factors, or the reality that people with epilepsy, especially those with uncontrolled seizures, are living at the lowest household income levels and often have trouble getting the care they need when compared with the general population (10, 11). Assistance combating barriers of care via outpatient social services support has traditionally been provided via primary care offices and not specialty clinics. For some complex, chronic conditions, patients may be followed more closely by their specialty clinic than their primary care office. CHWs can serve to fill the gaps present in social and health care systems by serving as intermediaries between patients, their care teams, and community resources, thereby helping patients with problems that clinicians may not be able to address within the scope of a routine clinical visit (12–15). CHWs integrated onto a clinical center team can work with patients to clarify provider directives, assist with treatment program compliance, support patients in learning about their illness, review lifestyle management imperatives, assist with meeting transportation, housing, food security and insurance needs, and provide critical feedback to medical teams to assist with the tailoring of care plans (10, 16–18).

Furthermore, interventions involving CHWs have demonstrated to have a positive effect on physical health behaviors and outcomes in racial and ethnic minority communities, and for persons who have lacked access to necessary health care (19–21). Studies suggest CHW interventions can address these unmet needs, and demonstrate a positive financial return on investment in doing so (22).

Initiatives to increase opportunities for CHWs to train specifically in epilepsy have been targeted in recent population health efforts and include the Managing Epilepsy Well Community Health Worker Curriculum guide^1^ available for download online. Further, the availability of virtual modular CHW epilepsy training developed, piloted^2^ and now readily through the Community Epilepsy and Self-Management Training Center at Dartmouth Health^3^ has demonstrated widespread appeal to a spectrum of professionals working in CHW roles and consistently high user satisfaction with the educational content provided through the accredited training. Both CHW training opportunities are readily accessible and present a pathway for the expansion of a CHW epilepsy knowledgeable workforce to serve patients at epilepsy centers and in their diverse communities.

Understanding clinician and patient readiness to integrate CHWs into the epilepsy center setting can inform and facilitate successful and sustainable integration. The multidisciplinary composition of most epilepsy center teams suggests that only a subset of team members will have had prior experience and knowledge of CHWs and their work in the health care system. There may be more acceptance or appreciation for addressing SDOH, and thus CHWs integration, among nursing and social work staff who are currently addressing these gaps for patients at their center. Clinician perspectives will shape CHW utilization, referrals, and collaboration with the epilepsy team, and will influence the partnerships created with their patients and CHWs. The current study objectives were two-fold: (1) to assess the readiness of epilepsy center clinical providers to integrate CHWs onto epilepsy clinical care teams; and (2) to gather insights from patients on their readiness to receive care from a CHW positioned at an epilepsy center.

## 2 Materials and methods

### 2.1 Survey development

We conducted a cross-sectional study at epilepsy centers across the New England region (Maine, Massachusetts, New Hampshire, New York, Rhodes Island and Vermont) between October 2021 and July of 2022. We designed online digital surveys using the Research Electronic Data Capture (REDCap) (23, 24) digital survey tool to collect voluntary self-reported participant survey data for two groups: clinicians and patients with epilepsy. All survey questions were multiple choice, most in Likert-style scale form, without a required response.

The development of the clinical provider survey and patient survey was guided by an expert panel of multidisciplinary epilepsy center clinical providers, CHW training program instructors and supervisors, and patients living with epilepsy. Survey questions were clustered into key thematic areas pertaining to working with and addressing clinic needs via a CHW at an epilepsy center: (1) prior knowledge and experience, (2) recruitment and selection, (3) roles and responsibilities, (4) training and supervision, (5) funding mechanisms, (6) epilepsy team/care gaps and challenges that impact patients most, (7) expectations and trust, and (8) center environment and collaborative culture. The provider survey included 70 items covering the above listed thematic areas, and the patient survey included 52 items with similar questions covering the areas of (1) prior knowledge and experience, (6) epilepsy team/care gaps and challenges that impact patients most, (7) expectations and trust, and (8) center environment and collaborative culture.

### 2.2 Setting and participants

We obtained a list of 12 New England regional division directors and contact emails and telephone numbers from a published National Association of Epilepsy (NAEC) digital registry. Study investigators contacted epilepsy division directors in an email message describing the project and inviting them to distribute the survey invitation to all center providers who met the study informant criteria. This included physicians, nurses, and social workers who met the following criteria: representing a clinical provider role working closely with people with epilepsy or being currently employed in a position involving services or studies related to epilepsy patients. In the invitation, clinicians were asked to fill out a one-time specialty-specific voluntary electronic survey on their attitudes and perceptions regarding CHW integration in their epilepsy center. Approximately 8 weeks after the initial email, epilepsy center leaders were emailed a second time and requested to forward the invitation again to bolster response rates. Patient surveys were collected only at one epilepsy center (Dartmouth Health Epilepsy Center).

### 2.3 Study procedures

All study procedures were approved by the University of Massachusetts Medical Center and Dartmouth Health Institutional Review Boards. The development and conduct of the research were guided by an expert panel of epilepsy clinicians and public health professionals.

### 2.4 Statistical analysis

Knowledge and attitudes about patient SDOH needs are presented as proportions for all providers combined. We also examined the distribution of knowledge and attitudes about community health workers by provider group. Because of the small number of nurses and social workers, we combined these two groups and compared them to physicians. Differences in knowledge and attitudes by provider groups (providers vs. nurses/licensed social workers) for dichotomous items were quantified using a Fisher’s Exact test because of small cell numbers. Between group differences in 5-point Likert-scale items were tested using a Wilcoxon Rank Sum test. Patient report of care gaps in SDOH were reported as the number of responses for each item. All analyses were conducted using Stata/MP version 16.1 (25).

## 3 Results

### 3.1 Clinician respondents

In total 12 epilepsy center directors from 12 NAEC epilepsy centers who were contacted by email communication participated in survey distribution (participation rate of 100%). Respondents (*n* = 65) were classified into the following categories, physicians (58%), nurses (20%), social workers (8%), neuropsychologists (6%) and epilepsy fellows (5%). Most were 25–44 years old and averaged 5–10 years of work in the field (Supplementary Table 1). Most respondents worked in a practice model that included a multidisciplinary team.

### 3.2 Prior CHW knowledge and experience

Between 23 and 38% of respondents reported having extensive knowledge of, or experience with, the role of CHWs in epilepsy care. Almost one-quarter (23%) of respondents indicated no knowledge of the role of CHWs in patient care. While 37% shared firsthand experience with CHWs, there was a general unawareness of the role a CHW serves in management of chronic disease in a health care system and the scientific evidence supporting it. Most respondents reported receiving information about CHWs from colleagues or in hospital meetings. More than three quarters (82.9%) of respondents disagree that their residents and fellow trainees receive the appropriate education on SDOH in epilepsy. The vast majority of respondents (88%) were not aware of a funding mechanism to support a CHW at epilepsy centers.

### 3.3 CHW recruitment and roles

A quarter of respondents reported having a CHW employed at their epilepsy center. These providers reported there were staff with knowledge and support at their medical center to recruit CHWs for the epilepsy clinic. Factors that made providers most comfortable with integrating a CHW into their epilepsy team included experience in mental health education and treatment, experience in health education and epilepsy and self-management CHW training. The perceived most essential qualities of a CHW reported were (1) self-directed, independent worker, committed and persistent, (2) connection and desire to help community service, and (3) ability to work in a multidisciplinary team and collaborate with caregivers. Over three-quarters (77%) of respondents felt a CHW for their epilepsy center would best be recruited from a community-based organization. For respondents with and without a CHW at their epilepsy center, the majority agreed that CHWs could provide culturally appropriate health education, information and outreach in a community-based setting, bridge and culturally mediate between individuals, communities and health and human services, assist patient in accessing the service and resources they need, provide direct services, such as counseling, social support, care coordination and health screenings, and can advocate for individual and community needs. They also agreed that it would be possible for the roles and responsibilities of a CHW to be clearly delineated and there could be a system in place to identify and address situations where additional team support is needed. Additionally, providers overwhelmingly agreed that CHWs could play a role in educating the members of an epilepsy center team about community-based supports and resources available to people with epilepsy.

### 3.4 CHW training and supervision

The majority (more than 85%) of respondents were unfamiliar with the availability of any CHW training program in their state or with the Centers for Disease Control CHW Epilepsy Self-Management program. Of epilepsy centers with CHWs, only 14% indicated that their CHW had completed accredited *Epilepsy and Self-Management Training* (26), and none had general CHW certification training or state accreditation.

Social worker, nurse and physician were the most common responses for who would be best to supervise a CHW hired to the epilepsy center. Almost half of respondents (41%) felt they would be able to provide sufficient supervision and guidelines to ensure that a CHW is providing the appropriate level of nonclinical care to epilepsy patients. Only a few respondents (13%) felt there was an effective system that could be used to track patient referrals to a CHW and their ability to meet patient needs.

### 3.5 Epilepsy team care gaps and challenges impacting patients

In terms of perceived care gaps, respondents reported that employment/unemployment filing for Supplemental Security Income disability, behavioral health, transportation, financial assistance, and housing were the top five needs of patients that required additional support not currently available through their epilepsy center. Additionally, seizure tracking, medication adherence, self-management and comorbidities were patient health education topics identified by respondents.

**Figure 1:**
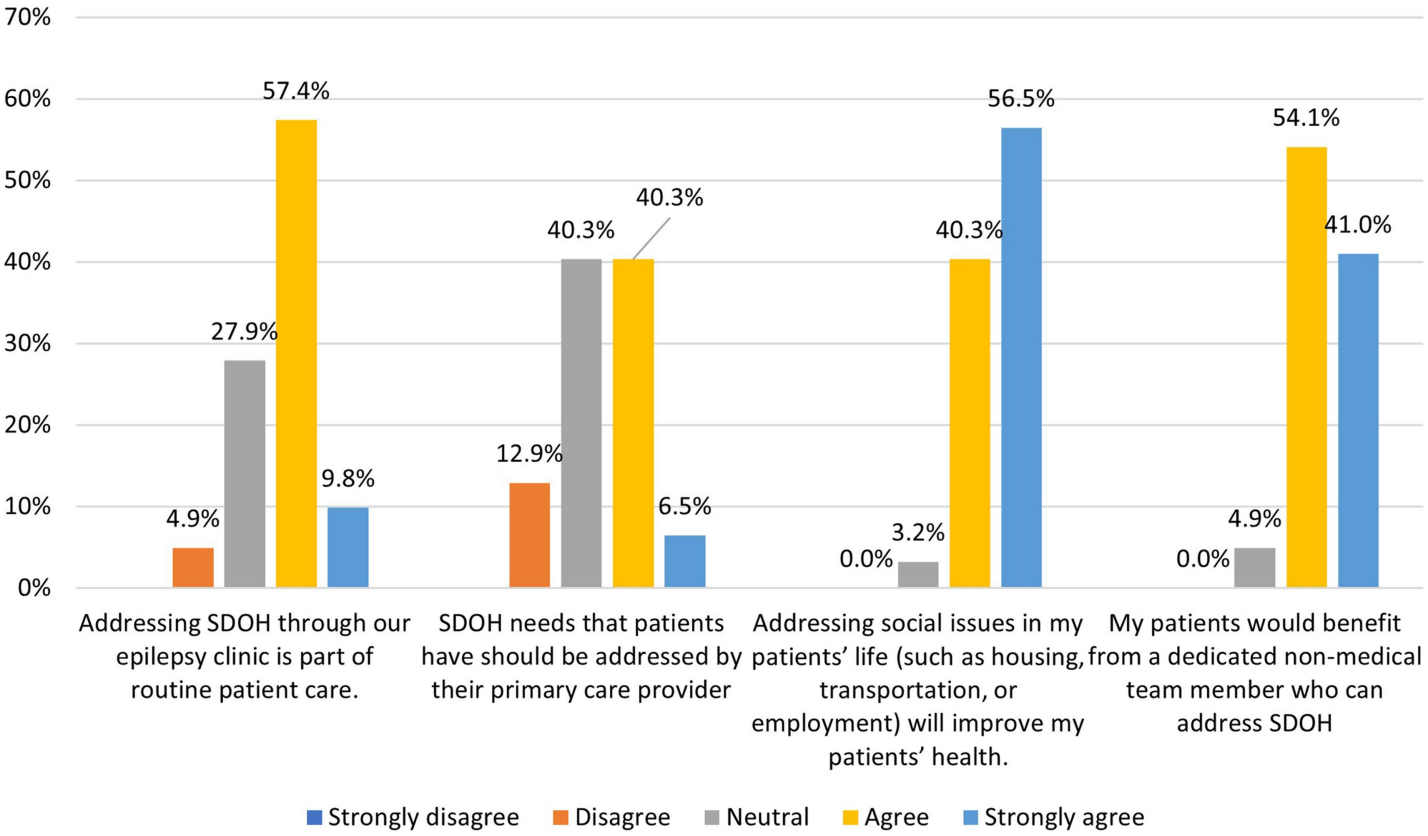
Clinician respondents (*n* = 65) perceptions of addressing SDOH gaps in clinic patients.

**Figure 2:**
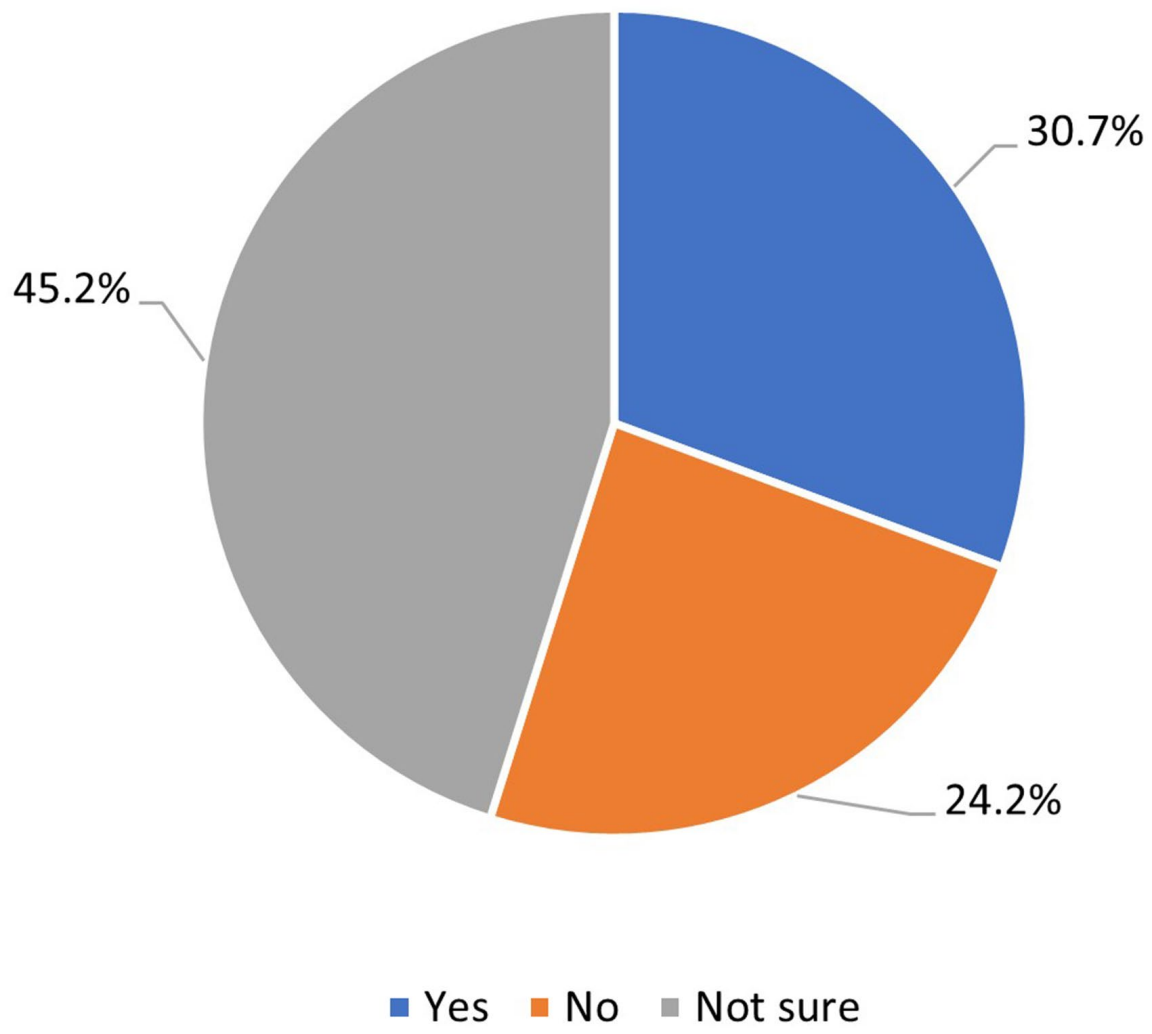
Clinician respondents (*n* = 65) awareness that patient’s SDOH needs are being address outside of the epilepsy center.

Almost all respondents indicated that addressing SDOH would benefit patients’ health (see Supplementary Table 2 for a list of identified needs). While 46% indicated that patients’ SDOH needs should be addressed by their primary care provider office, only 30% of respondents report being aware that their patients receive assistance with SDOH of their routine epilepsy team care at the epilepsy center (Figures 1, 2). Two thirds of respondents reported addressing SDOH through their epilepsy clinic as part of routine patient care, with social work and nursing being the current staff members who take on the role of screening for and addressing SDOH gaps in care at their epilepsy center. Nearly all (95%) responded they would refer patients to an epilepsy clinic based CHW and agreed their patients would benefit from a dedicated nonmedical team member who could address SDOH.

### 3.6 Provider expectations and center environment

**Figure 3:**
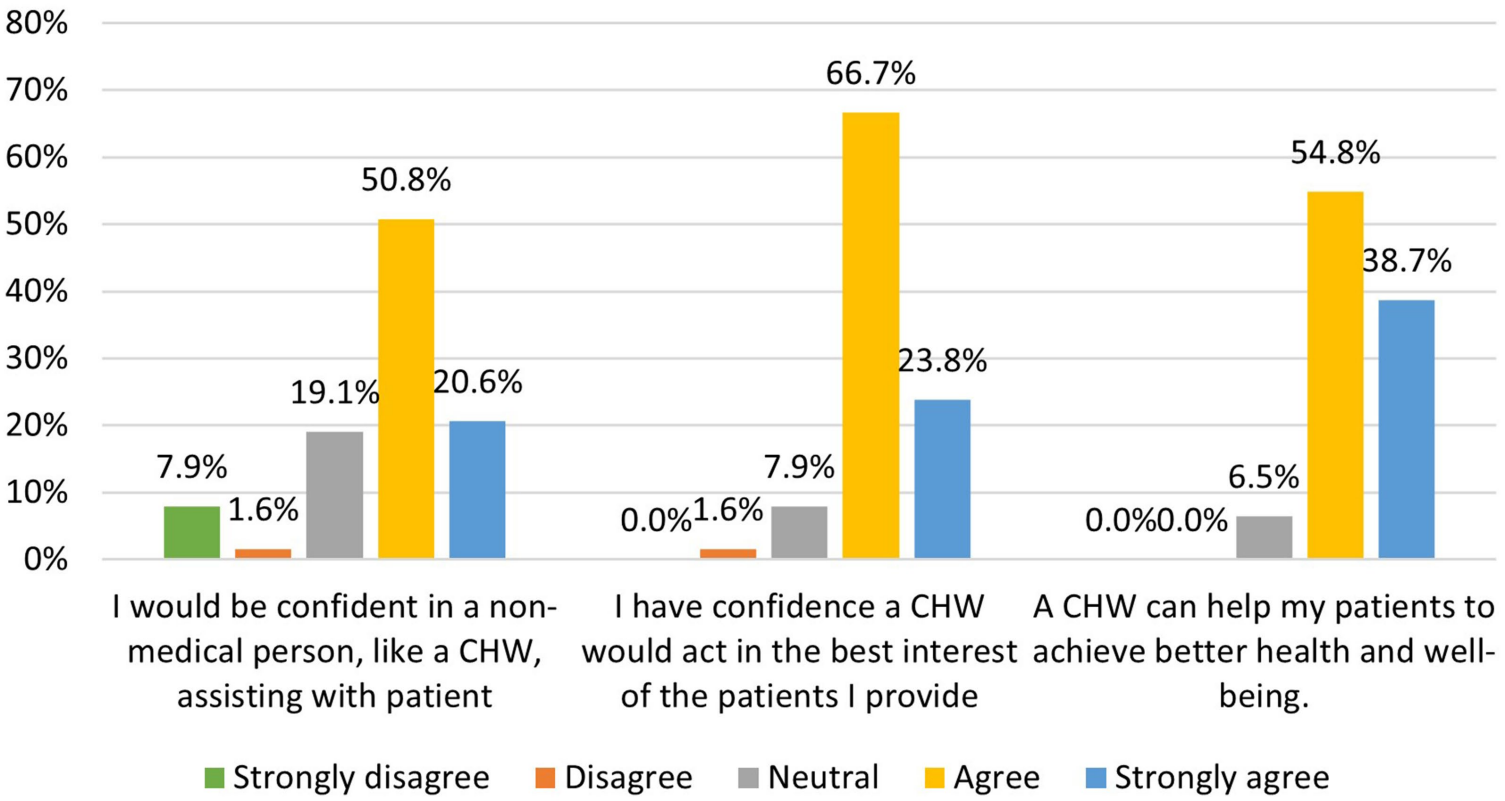
Clinician respondents (*n* = 65) perceptions of addressing SDOH gaps in clinic patients.

Most respondents saw a benefit to integrating a CHW onto their epilepsy team (Figure 3) and felt they would be confident in a CHW handling sensitive health, financial or other personal patient information. Providers indicated that epilepsy-specific training would boost their confidence in CHW’s ability to benefit patients. To best facilitate incorporation and acceptance into the healthcare team, 78% indicated that a CHW should be based in the clinic at the epilepsy center and 84% felt that CHWs should attend epilepsy team group meetings (e.g., nursing rounds). Additionally, 85% agreed that a mechanism (e.g., electronic record integration, weekly rounds) for CHWs to provide feedback to clinical providers on the epilepsy center team about patients whose care they participate in is necessary to achieve the highest level of patient care. Reported feedback given to providers by CHWs included information on high-risk patient populations, and socially and economically disadvantaged patients in the clinic.

Most respondents felt that CHWs would be capable of delivering standardized epilepsy self-management programs to patients if they were trained to do so, and about half of respondents thought it would be possible for a member of their epilepsy team to effectively supervise a CHW. Furthermore, 95% would refer their patients to a CHW if one were available in their epilepsy center and 93% thought that their epilepsy patients would welcome support from a CHW.

### 3.7 Physician versus nurse and social worker responses

**Figure 4:**
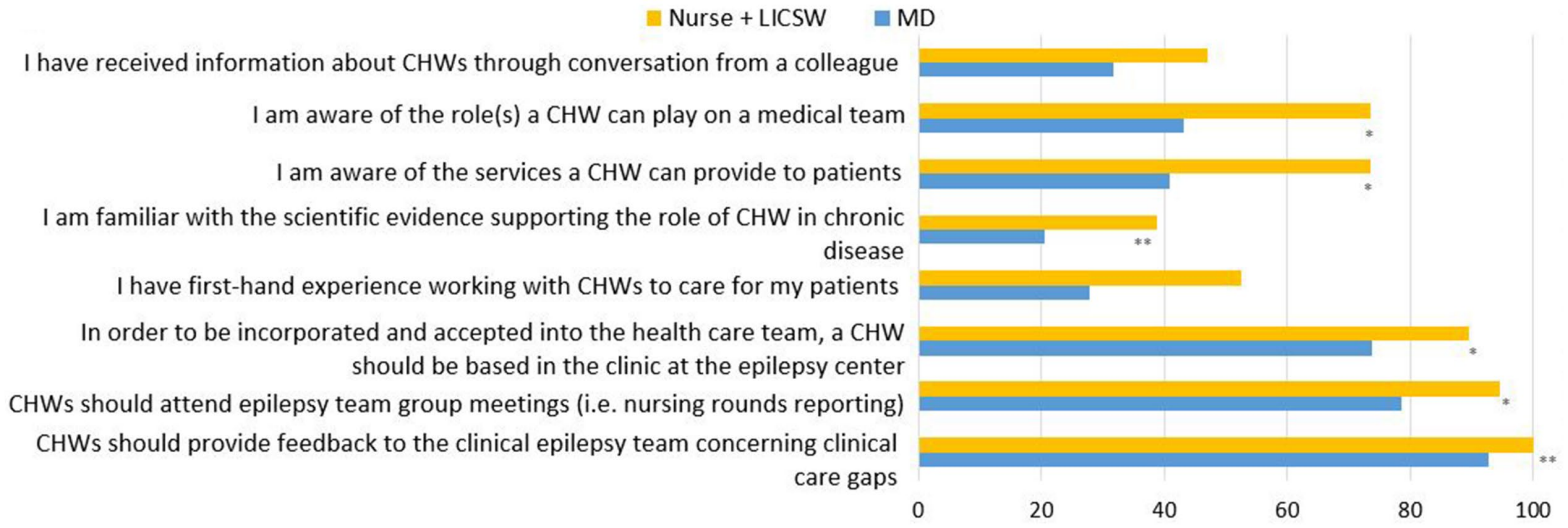
Distribution of knowledge and attitudes regarding the roles of CHWs and their integration in epilepsy care by item and provider type by percentage of affirmative answers. All “yes” vs. “no,” “strongly agree/agree” coded as affirmative; “neutral/disagree/strongly disagree” items coded as not affirmative. * *p* < 0.05, ** *p* < 0.01 Significant difference calculated by Wilcox Rank Sum.

Survey responses from physicians (*n* = 42) compared to nurses and licensed social workers (*n* = 19) were similar with the exception of physicians expressing less awareness of the role a CHW could play on a medical team, the services a CHW could provide to patients, and scientific evidence supporting the role of CHW in chronic disease (Figure 4). In contrast, there were no statistically significant differences in clinician perception of addressing SDOH gaps between physicians and nurses/licensed social workers.

Main differences between physicians and nurse + social workers were detected in questions regarding the level of involvement of the CHW in the clinical care of the patients. Physicians expressed less need for CHWs to be based at epilepsy center clinics and less need for them to attend epilepsy team group meetings. There was also less agreement among physicians when compared with nurses/social workers regarding CHWs providing feedback to the clinical epilepsy team concerning clinical care gaps.

### 3.8 Patient respondents

Patient participants (*n* = 21; all diagnosed with epilepsy; 61.9% female, 95.6% White, 95.25% not Hispanic or Latino; see Supplementary Table 3) accessed the survey via QR code and completed the survey on their smartphone. The survey included questions about whether or not participants were familiar with a CHW role or had previously received care from a CHW, whether or not participants would be willing to receive help from a CHW, participants’ confidence in a CHW addressing their identified needs, and whether or not participants believed a CHW could improve their health and well-being.

**Figure 5:**
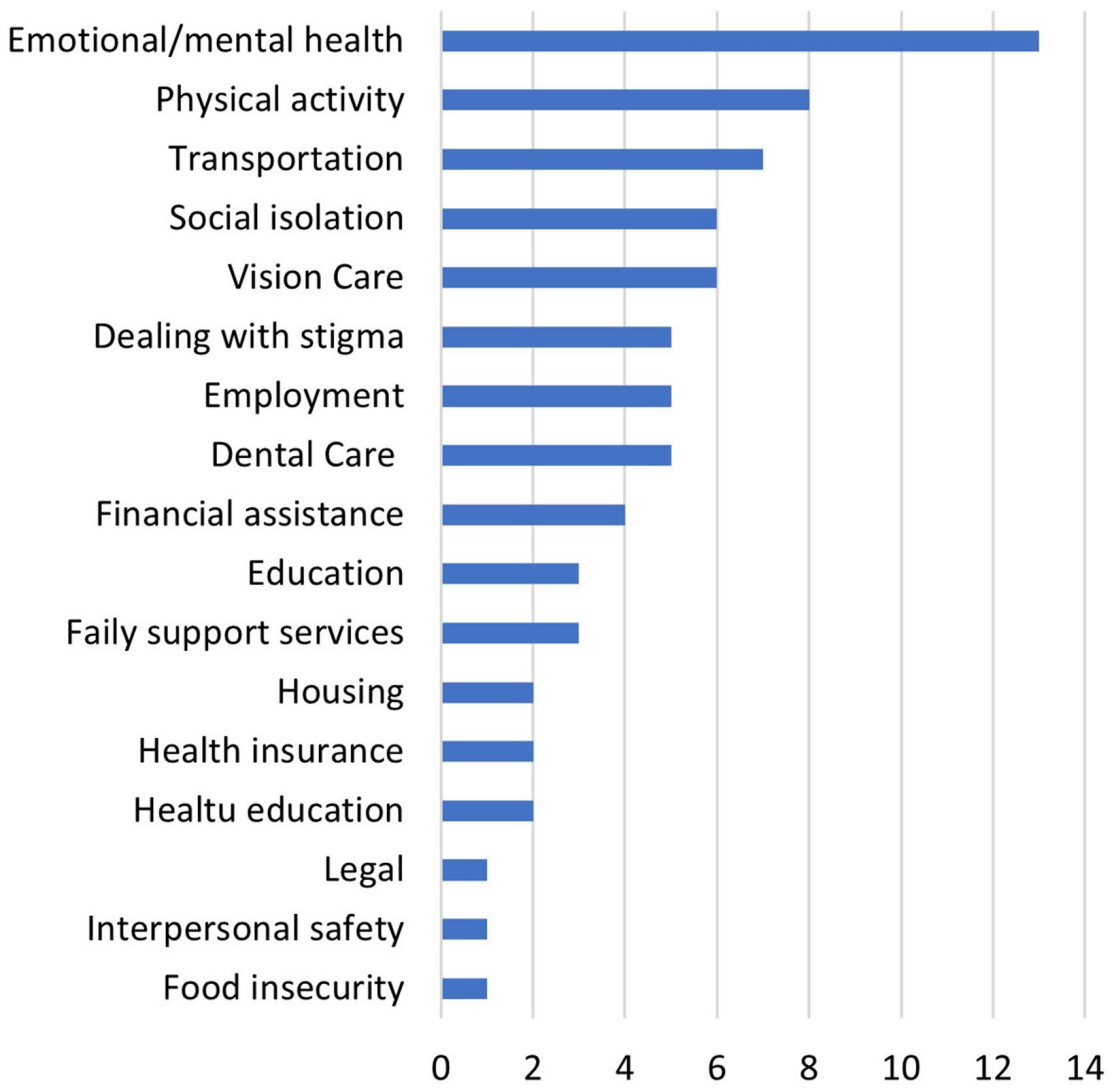
Patient respondents (*n* = 18) who listed their care gaps in specific SDOH.

Survey participants’ baseline knowledge of CHWs was divided (42.8% with prior knowledge and 42.8% without, 14.2% not sure). About 7 out of 10 (66.6%) of patient respondents indicated they had not received care from a CHW (19% said yes, 14.2% were unsure). When asked about prior experience receiving assistance meeting SDOH needs, 42.9% of respondents reported they had received help directly from their physician; 33.3% reported another member of the care team (CHW 14.2%, nurse 14.2%, social worker 4.76%), and 19% reported not ever having received this kind of assistance. Roughly three-quarters of respondents (71%) believed a CHW could connect them with local resources in their communities (4.7% disagreed, 19% were unsure), and 76% of participants reported being willing to receive epilepsy education from a CHW (9.52% said no, 14.2% were unsure). Overall, belief in a CHW’s ability to address patient needs was mixed, with 42.8% of respondents indicating that they were confident in that CHW’s ability (52.3% neutral, 4.76% disagreed). Nearly two-thirds of respondents (61.9%) of agreed that working with a CHW would improve their health and well-being (38.1% neutral). Although the small sample size prevents a correlation analysis, patients who expressed less confidence in a CHW’s ability to address their needs or their ability to improve health and well-being tended to be slightly older; otherwise, they did not seem demographically different from those who felt more confident in a CHWs ability to assist with care. The top four SDOH needs reported by patient respondents include support with emotional/mental health, physical activity, transportation and social isolation (Figure 5).

## 4 Discussion

This study’s contributions are unique in quantifying the responses of multidisciplinary epilepsy center clinicians regarding the readiness and potential for integrating CHWs onto epilepsy center care team. Within the New England region, few epilepsy clinicians have firsthand experience with CHWs and have limited understanding of their role as nontraditional health care professionals. Epilepsy clinicians are generally unaware of the scientific evidence showing positive effects of CHW health outcomes. Knowledge of CHW recruitment, training and supervision is low. Despite this, the data collected demonstrates that SDOH care gaps exist for epilepsy patients receiving care at epilepsy centers which could potentially be addressed by a nontraditional healthcare professional, such as a CHW, instead of a social worker or nurse, thus alleviating burden from advanced practice provider members to address other clinically based patient needs.

This survey identifies several potential opportunities to improve recruitment, training, and subsequent integration of a CHW into epilepsy center in the New England region. Broadening knowledge of the various roles and service a CHW can provide among providers will allow for better screening and referral of patients who have SDOH or other unmet needs. Ensuring epilepsy and self-management training, as well as (ideally) CHW certification, will standardize the quality of care and competency of CHWs in epilepsy centers. Introducing formal supervision, regular opportunities to provide feedback to the team, and tracking of CHW tasks will help ensure quality of care and clear communication among team members and patients. Our data demonstrate the lack of knowledge around establishing funding that could lead to sustainable roles for CHWs at an epilepsy center. Provider responses favor the potential benefits of a CHW integrated into an epilepsy center care team. Importantly, perceptions of care gap needs and benefits of integrating a CHW into the epilepsy team align between physicians, nurses and social workers but may diverge slightly as to how CHW should access and report back their progress with patients to the epilepsy team.

The patient cohort responding to the survey demonstrated a similar lack of knowledge or familiarity with CHWs and their role, indicating a need for not only educating providers about CHWs but also making information available to epilepsy patient communities around the potential role a CHW could play in their care. Our survey revealed a willingness to consider receiving education and other services from a CHW, but a lack of confidence in a CHW being able to help them meet their needs was present and may be a direct reflection about a lack of awareness of CHW roles. Despite the gap in confidence, more than half of patients felt working with a CHW could help to improve their overall health and well-being. Almost half of patients identified their physicians as being the person to have most often assisted them with SDOH needs, further bringing to light the burden on clinicians to address needs beyond medical decision making at a specialist office visit. Access to social workers is limited at most epilepsy centers given fiscal restraints and this leaves a window of opportunity to consider filling gaps in addressing SDOH with less costly non-traditional health professionals like CHWs.

### 4.1 Limitations

Possible limitations of our study are its cross-sectional design, reliance on self-reported data which is potentially subject to recall bias and reactivity to the assessment situation, and participant recruitment restricted to the Northeast possibly affecting the ability to generalize to broader populations. However, in a rapidly changing healthcare environment that reflects the increasingly diverse demographics in the United States, our findings are likely to be relevant beyond our study. This study was conducted during the COVID-19 pandemic, limiting participation numbers secondary to provider bandwidth and recruitment of patient participants.

## 5 Conclusion

The integration of CHWs onto epilepsy center teams presents an opportunity to augment the comprehensive care people with epilepsy receive at epilepsy centers by identifying and addressing SDOH, improving health literacy and communication between providers and patients. Despite limited understanding of CHW roles or firsthand experience with CHWs, clinician and patient readiness to consider integration of a CHW onto an epilepsy center team was high. The potential for benefit was generally endorsed by both clinicians and patients, and is evident in prior literature (10, 12, 16).

Further efforts are required to augment provider and patient knowledge of CHW role in specialty epilepsy care. Providing a model for integration of CHW onto epilepsy teams and sharing pathways supporting CHW funding and sustainability will improve efforts to address SDOH and outcomes for people with epilepsy.

## Data Availability

The raw data supporting the conclusions of this article will be made available by the authors, without undue reservation.

## Footnotes

^1^ https://managingepilepsywell.org/community-health-worker

^2^See Kiriakopoulos ET, Dawson T, Schmidt S, Jobst B. Pilot Implementation of Virtual Epilepsy and Self-Management Training for Community Health Workers. Abst. 2.369, Proceedings of the American Epilepsy Society Meeting, December, 2021.

^3^ https://www.dartmouth-hitchcock.org/hobscotch-institute/community-training
