# Supplementary material for: Clinician and patient readiness to engage with community health workers at epilepsy care centers
Source: Front Neurol. 2025 Apr 15;16:1580655. doi: 10.3389/fneur.2025.1580655 (PMC12037368; doi:10.3389/fneur.2025.1580655)
Supplement: Supplementary file 1 [file Table_1.docx]

**Supplementary Table 1.** Demographics of clinician survey takers (*n* = 65).

| ***n* %** | | | |
| --- | --- | --- | --- |
| ***Age Range*** | |  |  |
| 25–44 | | 31 | 47.7% |
| 45–54 | | 12 | 18.5% |
| 55–64 | | 13 | 20.0% |
| 65+ | | 6 | 9.2% |
| missing | | 3 | 4.6% |
| ***Workplace*** | |  |  |
| Community neurologist (not hospital-based) | | 4 | 6.2% |
| Epilepsy center | | 42 | 64.6% |
| Neurology at a hospital-based practice | | 17 | 26.2% |
| Other | | 2 | 3.1% |
| ***Percentage of practice dedicated to epilepsy*** | |  |  |
| < 10% | | 4 | 6.2% |
| > 90% | | 24 | 36.9% |
| 10–20% | | 11 | 16.9% |
| 20–50% | | 11 | 16.9% |
| 50–90% | | 15 | 23.1% |
| ***Current practice model*** |  |  |  |
| Physician and nurse combined care | | 10 | 15.4% |
| Physician-based care | | 12 | 18.5% |
| Team-based care (e.g., physician, fellow, nurse, social worker) | | 43 | 6.2% |
| ***Number of years caring for epilepsy patients*** | |  |  |
| < 5 | | 25 | 38.5 |
| > 20 | | 17 | 26.2% |
| 5–10 | | 10 | 15.4% |
| 10–20 | | 13 | 20.0% |

**Supplementary Table 2.** Clinician perception of patient SDOH needs.

| *Social Determinants of Health*   - Employment/Vocational support - Unemployment/Filing for SSI disability - Behavioral health - Transportation - Financial assistance - Housing | *Health Education*   - Seizure tracking - Medication adherence - Self-management - Comorbidities |
| --- | --- |

**Supplementary Table 3:** Demographics of people with epilepsy survey respondents (*n* = 21).

| ***n* %** | | | | | |
| --- | --- | --- | --- | --- | --- |
| ***Age Range*** | | | |  |  |
| 18–24 | | | | 2 | 0.10% |
| 25–34 | | | | 5 | 0.24% |
| 35–44 | | | | 4 | 0.19% |
| 45–54 | | | | 3 | 0.14% |
| 55–64 | | | | 3 | 0.14% |
| 65+ | | | | 4 | 0.19% |
| ***Gender*** | | | |  |  |
| Female | | | | 12 | 0.57% |
| Male | | | | 7 | 0.33% |
| Non-binary/third gender | | | | 0 | 0.00% |
| Prefer not to say | | | | 1 | 0.05% |
| Other | | | | 1 | 0.05% |
| ***Race*** | | | |  |  |
| Black or African American | | | | 0 | 0.00% |
| American Indian or Alaska Native | | | | 1 | 0.05% |
| Asian: Chinese, Filipino, Japanese, Korean, Asian Indian or Thai | | | | 0 | 0.00% |
| Asian (other than above) | | | | 0 | 0.00% |
| Native Hawaiian or Pacific Islander | | | | 0 | 0.00% |
| White | | | | 19 | 0.90% |
| Unknown | | | | 0 | 0.00% |
| More than one race | | | | 0 | 0.00% |
| Prefer not to answer | | | | 1 | 0.05% |
| ***Ethnicity*** | | | |  |  |
| Hispanic or Latino | | | | 1 | 0.05% |
| Not Hispanic or Latino | | | | 17 | 0.81% |
| Prefer not to answer | | | | 3 | 0.14% |
| ***Primary language spoken in home*** | | | |  |  |
| English | | | | 21 | 1.00% |
| ***Highest level of education*** | | | |  |  |
| High school/GED | | | | 4 | 0.19% |
| Some college | | | | 3 | 0.14% |
| 2-year college degree | | | | 6 | 0.29% |
| 4-year college degree | | | | 5 | 0.24% |
| Master’s degree | | | | 3 | 0.14% |
| ***Marital status*** |  |  |  |  |  |
| Married | | | | 7 | 0.33% |
| Never married | | | | 8 | 0.38% |
| A member of an unmarried couple | | | | 1 | 0.05% |
| Divorced | | | | 4 | 0.19% |
| Widowed | | | | 1 | 0.05% |
| ***Current employment status*** | | | |  |  |
| Working full-time | | | | 10 | 0.48% |
| Working part-time | | | | 3 | 0.14% |
| Disabled | | | | 4 | 0.19% |
| ***n* %** | | | | | |
| Retired | | | | 3 | 0.14% |
| Homemaker | | | | 1 | 0.05% |
| Unemployed | | | | 0 | 0.00% |
| ***Current housing situation*** | | | |  |  |
| House | | | | 14 | 0.67% |
| Apartment | | | | 6 | 0.29% |
| Manufactured/mobile home | | | | 1 | 0.05% |
| ***Current health insurance status*** | | | |  |  |
| Insured by Medicaid | | | | 5 | 0.24% |
| Insured by Medicare | | | | 5 | 0.24% |
| Insured by a private insurance company | | | | 10 | 0.48% |
| Insured, but not sure what type | | | | 1 | 0.05% |
| Not insured | | | | 0 | 0.00% |
