# Supplementary material for: Clinician and patient readiness to engage with community health workers at epilepsy care centers
Source: Front Neurol. 2025 Apr 15;16:1580655. doi: 10.3389/fneur.2025.1580655 (PMC12037368; doi:10.3389/fneur.2025.1580655)
Supplement: Supplementary file 2 [file Table_2.docx]

**PATIENT OPINION SURVEY – COMMUNITY HEALTH WORKERS FOR EPILEPSY CARE**

We’re interested in your opinions about how a Community Health Worker (CHW) can help people with epilepsy reach their health goals and meet their social needs.

Epilepsy researchers and health providers from the University of Massachusetts Prevention Research Center and the Epilepsy Centers at the University of Massachusetts Medical Center and the Dartmouth-Hitchcock Medical Center in New Hampshire developed this survey.

If you don’t know much about community-health workers, we have included some background information about them to help you consider different ways they can be helpful in the care of people with epilepsy.

Your survey responses are anonymous and confidential.

The survey will take about 15-20 minutes to complete. Your participation in this survey is voluntary and you do not have to answer any questions you do not feel comfortable with.

If you have any questions about the survey please email

By clicking the button below, you acknowledge that your participation in the survey is voluntary, you are 18 years of age or older, and that you are aware that you may choose to end your participation in the survey at any time and for any reason.

Before beginning the survey, we would like to share with you the following information about Community Health Workers. Sometimes Community Health Workers are also referred to as Community Health Educators, Patient Navigators or Community Resource Specialists.

Who is a community health worker (CHW)?

The Massachusetts Department of Public Health defines a Community Health Workers (CHW) this way:

CHWs are public health workers who apply their unique understanding of the experience, language and/or culture of the populations they serve in order to carry out one or more of the following roles:

- Providing culturally appropriate health education, information and outreach in community-based settings, such as homes, schools, clinics, shelters, local businesses and community centers
- Bridging and/or culturally mediating between individuals, communities and health and human services, including actively building individual and community capacity
- Assisting people to access the services they need
- Providing direct services, such as informal counseling, social support, care coordination and health screenings
- Advocating for individual and community needs

CHWs are distinguished from other health professionals because they:

- Are hired primarily for their understanding of the populations and communities they serve
- Spend a significant portion of time conducting outreach in the categories above
- Have experience providing services in community settings

Finally, CHWs have been used for many years in the care of people living with chronic conditions such as diabetes, asthma, cancer, and heart disease, and other conditions.

These first questions will ask about your previous knowledge about CHWs.

1. **Have you heard of a Community Health Worker (CHW) before taking this survey? They are also known as a Community Health Educator, Patient Navigator or Community Resource Specialist.**

Yes

No

Not sure

2. **If yes, please indicate how well you feel you understand their role in helping patients with chronic diseases and what services they provide:**

| Not at all | A little bit | Somewhat | Quite a bit | Completely |
| --- | --- | --- | --- | --- |

3. **Have you ever had a CHW help you in the treatment of your epilepsy or another condition? (check all that apply)**

Yes, at the epilepsy center

Yes, in another healthcare setting

No

Not sure

**Please indicate how much you agree with each of the following statements. If you are a person with epilepsy answer the questions for yourself. If you are a caregiver of a person with epilepsy, think about the needs of your loved one when answering the question.**

4. **A CHW can help me (or family member with epilepsy) navigate the healthcare and other systems.**

| Strongly disagree | Disagree | Neutral | Agree | Strongly agree |
| --- | --- | --- | --- | --- |

5. **A CHW can connect me (or family member with epilepsy) with resources in the local community.**

| Strongly disagree | Disagree | Neutral | Agree | Strongly agree |
| --- | --- | --- | --- | --- |

6. **A CHW who can help** **me (or family member with epilepsy) with social needs (such as housing, transportation, or employment) would improve my (or family member with epilepsy) quality of life.**

| Strongly disagree | Disagree | Neutral | Agree | Strongly agree |
| --- | --- | --- | --- | --- |

Now, we’re going to ask you about your experiences with your epilepsy center team.

1. **Please select ALL of the following areas which you would like help with:**

| Child care | Health education (including epilepsy education)  Health education about other conditions |
| --- | --- |
| Emotional/mental health | Health insurance |
| Family support services | Housing |
| Dental care  Vision care (*add box)* | Interpersonal safety |
| Education | Legal |
| Employment | Medical home |
| Financial assistance | Transportation |
| Food insecurity | Other |
| Social isolation/Connecting with others | Physical activity |
|  | Dealing with stigma |

Please indicate other: ______________________________________________________________

2. **Have you ever been asked about these issues at the epilepsy center where you currently receive care (either asked directly by a staff member or by completing a survey)?**

Yes

No

Not sure

3. **The epilepsy center where I currently receive care provides assistance in addressing these problems:**

| Strongly disagree | Disagree | Neutral | Agree | Strongly agree |
| --- | --- | --- | --- | --- |

4. **The epilepsy team member who has helped to provide this assistance is:**

CHW

Nurse

Social worker

Physician

☐ Administrative staff (PCA

Other staff member: _______­­­­­­­­­­­­­­­­­­­­­­­­­­­­­­­­­­­­­­­­­­­­­­­­­­­­­­­­­

N/A _I disagreed that I got help in these areas.

Now, we’d like you to think about the opportunity to have a CHW as part of your (or your loved one’s) epilepsy team. We’ll ask you what you think about working with a CHW.

I. **I would be** **willing to allow a CHW to help me address needs that impact my (my loved one’s) health and well-being** *(examples: transportation, insurance, food, housing, unemployment, financial assistance)*

| Definitely not | Probably not | Not sure | Probably | Definitely |
| --- | --- | --- | --- | --- |

2. **I would** **be willing to receive epilepsy education from a CHW.**

| Definitely not | Probably not | Not sure | Probably | Definitely |
| --- | --- | --- | --- | --- |

3. **I would be willing to let a CHW help me (or my loved one) navigate the healthcare system? (examples: helping you make appointments, communicate with providers, help with medication refills)**

| Definitely not | Probably not | Not sure | Probably | Definitely |
| --- | --- | --- | --- | --- |

4. **I would** f**eel comfortable having a CHW sit in on my (or my loved one’s) medical appointments to help me keep track of what my doctor or nurse explained.**

| Definitely not | Probably not | Not sure | Probably | Definitely |
| --- | --- | --- | --- | --- |

5. **I would be comfortable sharing personal information with a CHW (which may include information about my health, employment, or health insurance):**

| Definitely not | Probably not | Not sure | Probably | Definitely |
| --- | --- | --- | --- | --- |

6. **I am comfortable working with a CHW over several weeks or months in order to address an issue I need help with**

| Definitely not | Probably not | Not sure | Probably | Definitely |
| --- | --- | --- | --- | --- |

7. **How would you prefer to interact with a CHW? (check all that apply)**

In-person at the epilepsy center

In-person in the community

In-person in my home

In-person in my community

On webcam (e.g., Zoom)

On the telephone

Via email or other written communication

Because we are in the middle of the COVID pandemic and this may go on for several more months, we’d like to ask you a few questions about this.

1. **As we continue to implement COVID-prevention measures during the pandemic, please rate where and how you would prefer to meet with a CHW.**

**At home by phone**

| Strongly disagree | Disagree | Neutral | Agree | Strongly agree |
| --- | --- | --- | --- | --- |

**At home online**

| Strongly disagree | Disagree | Neutral | Agree | Strongly agree |
| --- | --- | --- | --- | --- |

**At home face-to-face in a home visit**

| Strongly disagree | Disagree | Neutral | Agree | Strongly agree |
| --- | --- | --- | --- | --- |

**In the clinic**

| Strongly disagree | Disagree | Neutral | Agree | Strongly agree |
| --- | --- | --- | --- | --- |

**In my community**

| Strongly disagree | Disagree | Neutral | Agree | Strongly agree |
| --- | --- | --- | --- | --- |

2. **After the pandemic has ended and it’s safe to be around other people, please rate where and how you would prefer to meet with a CHW.**

**At home by phone**

| Strongly disagree | Disagree | Neutral | Agree | Strongly agree |
| --- | --- | --- | --- | --- |

**At home online**

| Strongly disagree | Disagree | Neutral | Agree | Strongly agree |
| --- | --- | --- | --- | --- |

**At home face-to-face in a home visit**

| Strongly disagree | Disagree | Neutral | Agree | Strongly agree |
| --- | --- | --- | --- | --- |

**In the clinic**

| Strongly disagree | Disagree | Neutral | Agree | Strongly agree |
| --- | --- | --- | --- | --- |

**In my community**

| Strongly disagree | Disagree | Neutral | Agree | Strongly agree |
| --- | --- | --- | --- | --- |

These questions will ask you how confident you are in the ability of CHWs to help you with your epilepsy care.

1. **Please indicate how much you agree with each of the following statements:**

**I feel confident about the abilities of a CHW to address my social needs.**

| Strongly disagree | Disagree | Neutral | Agree | Strongly agree |
| --- | --- | --- | --- | --- |

**I feel confident about the knowledge of CHWs to identify and connect me with the right resources in my community.**

| Strongly disagree | Disagree | Neutral | Agree | Strongly agree |
| --- | --- | --- | --- | --- |

**I feel comfortable with a CHW communicating with my doctor (neurologist, epileptologist, primary care provider) about my health and needs.**

| Strongly disagree | Disagree | Neutral | Agree | Strongly agree |
| --- | --- | --- | --- | --- |

**A CHW will help me make good decisions regarding my health and needs.**

| Strongly disagree | Disagree | Neutral | Agree | Strongly agree |
| --- | --- | --- | --- | --- |

**CHWs do a good job taking care of people like me**.

| Strongly disagree | Disagree | Neutral | Agree | Strongly agree |
| --- | --- | --- | --- | --- |

**I trust that a CHW will act in my best interest.**

| Strongly disagree | Disagree | Neutral | Agree | Strongly agree |
| --- | --- | --- | --- | --- |

**Working with a CHW will improve my health and well-being.**

| Strongly disagree | Disagree | Neutral | Agree | Strongly agree |
| --- | --- | --- | --- | --- |
|  |  |  |  |  |

Finally, please answer a few questions about your background including your epilepsy.

1. **Please indicate which best describes you:**

I am completing this survey for myself – I am a person with epilepsy

I am completing this survey for someone else – A close loved one or someone I care for is a person with epilepsy

2. **What is your age?**

18-24

25-34

25-44

45-54

55-64

65+

3. **What is your gender?**

Female

Male

Non-binary/third gender

Prefer not to say

Other

4. **What city and state do you currently live in**? _______________________________

5. **What is your race? (please select one)**

Black or African American

American Indian or Alaska Native

Asian: Chinese, Filipino, Japanese, Korean, Asian Indian or Thai

Asian (other than above)

Native Hawaiian or Pacific Islander

White

Unknown

More than one race

Prefer not to answer

6.**What is your ethnicity? (please select one)**

Hispanic or Latino

Not Hispanic or Latino

Prefer not to answer

7. **What is the primary language spoken in your home?**

English

Spanish

Other: ___________

8. **Please indicate the highest level of education you have completed,**

- - High school/GED
  - Some college
  - 2-year college degree
  - 4-year college degree

Please indicate your field of study: ____________________________

- - Master’s degree

Please indicate your field of study: ____________________________

- - Doctoral degree

Please indicate your field of study: ____________________________

- - Professional degree (e.g., MD, JD)

9. **What is your current marital status?**

☐Married

☐Never married

☐A member of an unmarried couple

☐Divorced

☐Separated

☐Widowed

☐Prefer not to answer

10. **What is your current employment status?**

□ Working full-time

□ Working part-time

□ Working from home full-time

□ Working from home part-time

□ Full-time student

□ Disabled

□ Retired

□ Homemaker

□ Furloughed

□ Unemployed

11. **What is your current housing situation (the physical space in which you live)?**

□ House

□ Apartment

□ Manufactured/mobile home

□ Temporarily in a hotel or motel

□ Permanently in a hotel or motel

□ Rooming house or boarding house

□ Boat or other vehicle

□ Tent or outside

□ Other (please specify): _________

12. **What is your current health insurance status?**

Insured by Medicaid

Insured by Medicare

Insured by a private insurance company

Insured, but not sure what type

Not insured

Not sure/don’t know

13. **What type(s) of seizures do you have?**

- Focal Onset Aware
- Focal Onset Impaired Awareness
- Focal to Bilateral Tonic Clonic
- Generalized Onset
- Unknown Onset
- Don’t know/not sure

14. **I have seizures,**

- Every day
- Once a week
- More than once a week
- Every month
- More than once a month
- Every year
- More than once a year
- Every few years
- Never – my seizures are well controlled with treatment (medication, surgery, VNS, RNS, DBS, ketogenic diet)

15. **I was diagnosed with epilepsy age:**

- Newborn to 2 years old
- 3 to 12 years old
- 13 to 18 years old
- 18 to 54 years old
- 55 to 64 years old
- age 65 years or older

16. **At what epilepsy center do you currently receive care for your epilepsy?**

_______________________________________

17. **I receive my epilepsy medical care from (check all that apply),**

- Neurologist
- Epilepsy specialist (epileptologist)
- Epilepsy Nurse
- Nurse
- Physician Assistant
- Neuropsychologist
- My primary care provider (PCP)

18. **I receive additional support for my epilepsy from (check all that apply)**

- Social worker
- Counselor
- Other behavioral health provider
- Community organization (examples: Epilepsy Foundation, Epilepsy Association, CURE)

**Thank you for participating in this survey. The information you have shared**

**with us will be summarized, along with information provided by other**

**people who complete the survey. Individual names or other**

**indentifying information will not be included in any reports.**

*If you are interested in participating in an interview to share more thoughts on community health workers for epilepsy care please click on this link:
