# Supplementary material for: Clinician and patient readiness to engage with community health workers at epilepsy care centers
Source: Front Neurol. 2025 Apr 15;16:1580655. doi: 10.3389/fneur.2025.1580655 (PMC12037368; doi:10.3389/fneur.2025.1580655)
Supplement: Supplementary file 3 [file Table_3.docx]

**CLINICIAN SURVEY-COMMUNITY HEALTH WORKERS FOR EPILEPSY CARE**

This survey is being distributed by researchers from the University of Massachusetts Prevention Research Center and the Epilepsy Centers at the University of Massachusetts Medical Center and the Dartmouth-Hitchcock Medical Center in New Hampshire. The purpose of this survey is to help to better understand the readiness of epilepsy centers to integrate community health workers (CHWs) into their clinical teams to assist patients with epilepsy reach their health goals and meet their social needs. Survey responses will remain anonymous and confidential. The survey will take approximately 20-30 minutes to complete. Your participation in this survey is voluntary and you do not have to answer any questions you do not feel comfortable with.

If you would like to contact our research team to discuss this survey, please email Barbara Glidden, CCRP at Barbara.Glidden2@umassmemorial.org

By clicking the button below, you acknowledge that your participation in the survey is voluntary, you are 18 years of age or older, and that you are aware that you may choose to end your participation in the survey at any time and for any reason.

**The Massachusetts Department of Public Health definition of a Community Health Workers (CHW) is provided below. For the purpose of this survey the term CHW will encompass Patient Navigators, Community Health Navigators, Community Resource Specialists**

CHWs are public health workers who apply their unique understanding of the experience, language and/or culture of the populations they serve in order to carry out one or more of the following roles:

- Providing culturally appropriate health education, information and outreach in community-based settings, such as homes, schools, clinics, shelters, local businesses and community centers
- Bridging and/or culturally mediating between individuals, communities and health and human services, including actively building individual and community capacity
- Assisting people to access the services they need
- Providing direct services, such as informal counseling, social support, care coordination and health screenings
- Advocating for individual and community needs

CHWs are distinguished from other health professionals because they:

- Are hired primarily for their understanding of the populations and communities they serve
- Spend a significant portion of time conducting outreach in the categories above
- Have experience providing services in community setting

Thank you for participating in this survey.

**General Demographics**

**Please answer a few questions about your background and describe your involvement of care with epilepsy patients.**

1. What is your age?

18-24

25-44

45-54

55-64

65+

2. What is your gender?

Female

Male

Non-binary/third gender

Prefer not to say

Other

3. I am a,

Neurologist

Epileptologist (fellowship trained)

Epilepsy nurse specialist

RN

APRN

LPN

Physician Assistant

LICSW

Epilepsy fellow

4. Practice location,

MA

NH

VT

ME

RI

CT

5. Practice description

Epilepsy center

Neurology at a hospital-based practice

Community neurologist (not hospital based)

Other __________________________

6. Percentage of practice dedicated to epilepsy,

<10%

10 to 20%

20 to 50%

50 to 90%

> 90%

7. Current practice model is,

- Physician based care
- Physician and nurse combined care
- Team based care (e.g., physician, fellow, nurse, LICSW)

8. Number of years caring for epilepsy patients

- < 5
- 5-10
- 11-20
- >20

9. Check **all that apply**, our epilepsy clinical center team includes,

- Neurologists
- Epileptologists
- Epilepsy nurse specialists
- RN
- APRN
- LPN
- LICSW other social work/counseling
- PA
- Physician
- Neuoropsychologist
- Psychiatry
- CHW
- Fellows in training
- Residents in training

**These questions will ask about your prior knowledge about Community Health Workers (CHWs) (For the purpose of this survey the term CHW will encompass Patient Navigators, Community Health Navigators and Community Resource Specialists)**

10**. Does your *medical center* employ CHWs?**

- Yes
- No
- Not sure

11. **Does your *epilepsy center* employ CHWs?**

- Yes
- No
- Not sure

12. **I am aware of the role(s) a CHW can play on a medical team.**

| Strongly disagree | Disagree | Neutral | Agree | Strongly agree |
| --- | --- | --- | --- | --- |

13. **I am aware of the services a CHW can provide to patients.**

| Strongly disagree | Disagree | Neutral | Agree | Strongly agree |
| --- | --- | --- | --- | --- |

14. **I have firsthand experience working together with a CHW to care for my patients.**

- Yes
- No
- Not sure

15. **I am aware my patients receive assistance with social determinants of health from a CHW outside of their routine clinical epilepsy team care (not at your epilepsy center).**

- Yes
- No
- Not sure

16. **I am familiar with the scientific evidence supporting the role of CHW in chronic disease.**

| Strongly disagree | Disagree | Neutral | Agree | Strongly agree |
| --- | --- | --- | --- | --- |

17. **I am aware if the state I work in has training programs with accreditation for CHWs.**

Yes

No

18. **I am aware of the Centers for Disease Control and Prevention Epilepsy and Self-Management Training Guide for CHWs.**

Yes

No

19.  **I have received information about CHWs through conversation from a colleague.**

- Yes
- No

20. **I have received information about CHWs in a formal meeting setting from colleagues or hospital administrators.**

- Yes
- No

21. **I have attended a session on CHWs and epilepsy care at a professional conference.**

- Yes
- No

22. **My knowledge of CHW roles in patient care is,**

- Extensive
- Great
- Moderate
- Minimal
- None

23. **My knowledge of how to secure funding for a CHW is**,

- Extensive
- Great
- Moderate
- Minimal
- None

**CHW Recruitment and Selection**

**These next questions ask you to think about what the recruitment and hiring process would look like for a CHW and what experience and skills they should have.**

**YES (conditional from Q11: IF YES TO QUESTION 11)**

24. **How many CHWs are employed at your epilepsy center?**

- 1
- 2
- >2

25. **Our CHW(s) was recruited from (check all that apply),**

- Existing CHW program at our medical center/hospital
- Existing workforce at epilepsy center
- Community based organization
- State based CHW organization
- Family member or caregiver of an epilepsy patient
- Other: ____________________
- Don’t know

26. **There are staff with knowledge and support at my medical center to recruit CHWs for the epilepsy clinic.**

Definitely not Unlikely Unsure Likely Very likely

27. **I feel our epilepsy center team had/has the knowledge base required to construct a CHW job description and to interview candidates for a CHW** **role at our epilepsy center.**

| Strongly disagree | Disagree | Neutral | Agree | Strongly agree |
| --- | --- | --- | --- | --- |

28. **The following variable made me feel comfortable with integrating a CHW into our epilepsy team:**

***(Select top 3 and rank in the following order, 1=most important, 2=very important, 3-important)***

- - Experience in health education
  - State CHW Training or Certification
  - Epilepsy and Self-Management CHW Training
  - Licensure in a health profession
  - Strong volunteer experience working with epilepsy patients in the community
  - Interpreter certification and/or experience serving a multilingual population
  - Experience in mental health education and treatment
  - Clearance from our medical center based CHW program

29. **The most essential qualities of our CHW include:**

***(Select top 3 and rank in the following order, 1=most important, 2- very important, 3=important)***

- Connection and desire to help community served
- Cultural sensitivity, flexible, open-minded
- Self-directed, independent worker, committed and persistent
- Empathic, compassionate
- Constructive in interpersonal relationships and able to resolve conflict
- Creative, willingness to learn, resourceful
- Communication skills, bilingual
- Integrity, honesty and respectful
- Ability work in a multidisciplinary team and collaborate with caregivers

**NO (conditional from Q11: IF NO TO QUESTION 11)**

30**. A CHW for our epilepsy center would be recruited from,**

- Existing CHW organization
- Existing CHW at our medical center
- Existing workforce at epilepsy center
- Community based organization
- State based CHW organization
- Family member or caregiver of an epilepsy patient
- Other: ___________________________
- Don’t know/not sure

31. **There are staff with knowledge and support in place at my medical center to recruit CHWs for the epilepsy clinic.**

Definitely not Unlikely Unsure Likely Very likely

32. **I feel our epilepsy center team has the knowledge base required to construct a CHW job description and to interview candidates for a CHW** **role at our epilepsy center.**

| Strongly disagree | Disagree | Neutral | Agree | Strongly agree |
| --- | --- | --- | --- | --- |

33. **I am confident in my ability to interview potential CHW candidates and select a person to fill the role on our epilepsy center team.**

Not at all confident Slightly confident Moderately confident Very confident Extremely confident

34. **I would feel comfortable with a CHW integrating into our epilepsy team if they held the following:**

***(Select top 3 and rank them in the following order, 1=most important, 2- very important, 3=important)***

- - Experience in health education
  - State CHW Training or Certification
  - Epilepsy CHW Training
  - Licensure in a health profession
  - Strong volunteer experience working with epilepsy patients in the community
  - Interpreter certification and/or experience serving a multilingual population
  - Experience in mental health education and treatment
  - Clearance from our medical center based CHW program

35. **The essential qualities for a CHW to have, in order to be successful in providing care to epilepsy patients at our center include:**

**Connection to and desire to help community being served**

| Strongly disagree | Disagree | Neutral | Agree | Strongly agree |
| --- | --- | --- | --- | --- |

**Cultural sensitivity, flexible, open minded**

| Strongly disagree | Disagree | Neutral | Agree | Strongly agree |
| --- | --- | --- | --- | --- |

**Self-directed, independent worker, committed and persistent**

| Strongly disagree | Disagree | Neutral | Agree | Strongly agree |
| --- | --- | --- | --- | --- |

**Empathic, compassionate**

| Strongly disagree | Disagree | Neutral | Agree | Strongly agree |
| --- | --- | --- | --- | --- |

**Constructive in interpersonal relationships and able to resolve conflict**

| Strongly disagree | Disagree | Neutral | Agree | Strongly agree |
| --- | --- | --- | --- | --- |

**Creative, willingness to learn, resourceful**

| Strongly disagree | Disagree | Neutral | Agree | Strongly agree |
| --- | --- | --- | --- | --- |

**Communication skills, bilingual**

| Strongly disagree | Disagree | Neutral | Agree | Strongly agree |
| --- | --- | --- | --- | --- |

**Integrity, honesty and respectful**

| Strongly disagree | Disagree | Neutral | Agree | Strongly agree |
| --- | --- | --- | --- | --- |

**Ability work in a multidisciplinary team and collaborate with caregivers**

| Strongly disagree | Disagree | Neutral | Agree | Strongly agree |
| --- | --- | --- | --- | --- |

**ROLE AND RESPONSIBILITIES OF A CHW ON EPILEPSY CENTER TEAM**

**Because we are in the middle of the COVID pandemic and this may go on for several more months, we’d like to ask a few questions about this.**

**YES (conditional from Q11: IF YES TO QUESTION 11)**

36. **During the COVID-19 pandemic our CHW has interacted with patients via, *check all that apply*,**

- - Patient portal, electronic medical record
  - Telephone
  - Text message
  - Email
  - Online video one-on-one
  - Online video patient groups (e.g., support groups)
  - In-person, before or after clinician appointment
  - In-person, by separate appointment with CHW
  - In-person, CHW office hours
  - In-person, in patient groups (e.g., support groups)

37. **During the Covid-19 pandemic our CHW has interacted with the epilepsy team via, *check all that apply,***

- - Patient portal, electronic medical record
  - Faxed outpatient referral
  - Telephone
  - Text message
  - Email
  - Online video one-on-one
  - Online video patient groups (e.g. support groups)
  - Online video epilepsy multidisciplinary team meetings
  - In-person, before or after clinician appointment
  - In-person, by separate appointment with CHW
  - In-person, CHW office hours
  - In-person, in patient groups (e.g., support groups)
  - In-person, Epilepsy multidisciplinary team meetings

38. **Prior to the COVID-19 pandemic our CHW interacted with patients via, *check all that apply*,**

- - Patient portal, electronic medical record
  - Telephone
  - Text message
  - Email
  - Online video one-on-one
  - Online video patient groups (e.g. support groups)
  - In-person, before or after clinician appointment
  - In-person, by separate appointment with CHW
  - In-person, CHW office hours
  - In-person, in patient groups (e.g. support groups)

39. **Prior to the Covid-19 pandemic our CHW interacted with the epilepsy team via, *check all that apply,***

- - Patient portal, electronic medical record
  - Faxed outpatient referral
  - Telephone
  - Text message
  - Email
  - Zoom one-on-one
  - Online video patient groups (e.g. support groups)
  - Online video epilepsy multidisciplinary team meetings
  - In-person, before or after clinician appointment
  - In-person, by separate appointment with CHW
  - In-person, CHW office hours
  - In-person, in patient groups (e.g. support groups)
  - In-person, Epilepsy multidisciplinary team meetings

40. **Our CHW *provides culturally appropriate* health education, information and outreach in a community-based setting.**

| Strongly disagree | Disagree | Neutral | Agree | Strongly agree |
| --- | --- | --- | --- | --- |

41. **Our CHW *bridges and culturally mediates* between individuals, communities and health and human services.**

| Strongly disagree | Disagree | Neutral | Agree | Strongly agree |
| --- | --- | --- | --- | --- |

42. **Our CHW assists people to *access the services* they need.**

| Strongly disagree | Disagree | Neutral | Agree | Strongly agree |
| --- | --- | --- | --- | --- |

43. **Our CHW *provides direct services*, such as informal counseling, social support, care coordination and health screenings.**

| Strongly disagree | Disagree | Neutral | Agree | Strongly agree |
| --- | --- | --- | --- | --- |

44. **Our CHW *advocates* for individual and community needs.**

| Strongly disagree | Disagree | Neutral | Agree | Strongly agree |
| --- | --- | --- | --- | --- |
|  |  |  |  |  |

45. **The role and responsibilities of our CHW are clearly delineated.**

| Strongly disagree | Disagree | Neutral | Agree | Strongly agree |
| --- | --- | --- | --- | --- |

46. **There is a system in place to identify and address situations where additional team support is needed.**

| Strongly disagree | Disagree | Neutral | Agree | Strongly agree |
| --- | --- | --- | --- | --- |

**NO (conditional from Q11: IF NO TO QUESTION 11)**

47. **During the COVID-19 pandemic, a CHW would ideally interact with patients at our epilepsy center via: *(Check all that apply)***

- - Patient portal, electronic medical record
  - Telephone
  - Text message
  - Email
  - Online one-on-one
  - Online patient groups (e.g., support groups)
  - In-person, before or after clinician appointment
  - In-person, by separate appointment with CHW
  - In-person, CHW office hours
  - In-person, in patient groups (e.g., support groups)

48. **During the COVID-19 pandemic, a CHW would ideally interact with the epilepsy team via,**

***(Check all that apply)***

- - Patient portal, electronic medical record
  - Faxed outpatient referral
  - Telephone
  - Text message
  - Email
  - Online one-on-one
  - Online patient groups (e.g., support groups)
  - Zoom epilepsy multidisciplinary team meetings
  - In-person, before or after clinician appointment
  - In-person, by separate appointment with CHW
  - In-person, CHW office hours
  - In-person, in patient groups (e.g., support groups)
  - In-person, Epilepsy multidisciplinary team meetings

49. **In the absence of the COVID-19 pandemic, a CHW would ideally interact with patients at the epilepsy center via, check all that apply,**

- - Patient portal, electronic medical record
  - Faxed outpatient referral
  - Telephone
  - Text message
  - Email
  - Online one-on-one
  - Online patient groups (e.g. support groups)
  - Zoom epilepsy multidisciplinary team meetings
  - In-person, before or after clinician appointment
  - In-person, by separate appointment with CHW
  - In-person, CHW office hours
  - In-person, in patient groups (e.g. support groups)
  - In-person, Epilepsy multidisciplinary team meetings

50. **In the absence of the COVID-19 pandemic, a CHW would ideally interact with the epilepsy team via, check all that apply,**

- - Patient portal, electronic medical record
  - Faxed outpatient referral
  - Telephone
  - Text message
  - Email
  - Online one-on-one
  - Online patient groups (e.g., support groups)
  - Zoom epilepsy multidisciplinary team meetings
  - In-person, before or after clinician appointment
  - In-person, by separate appointment with CHW
  - In-person, CHW office hours
  - In-person, in patient groups (e.g., support groups)
  - In-person, Epilepsy multidisciplinary team meetings

51. **A CHW can *provide culturally appropriate* health education, information and outreach in a community-based setting.**

| Strongly disagree | Disagree | Neutral | Agree | Strongly agree |
| --- | --- | --- | --- | --- |

52. **A CHW can *bridge and/or culturally mediate* between individuals, communities and health and human services.**

| Strongly disagree | Disagree | Neutral | Agree | Strongly agree |
| --- | --- | --- | --- | --- |

53. **A CHW *can assist people to access* the services and resources they need.**

| Strongly disagree | Disagree | Neutral | Agree | Strongly agree |
| --- | --- | --- | --- | --- |

54. **A CHW *can provide direct services*, such as informal counseling, social support, care coordination and health screenings.**

| Strongly disagree | Disagree | Neutral | Agree | Strongly agree |
| --- | --- | --- | --- | --- |

55. **A CHW *can advocate* for individual and community needs.**

| Strongly disagree | Disagree | Neutral | Agree | Strongly agree |
| --- | --- | --- | --- | --- |
|  |  |  |  |  |

56. **It is possible for the roles and responsibilities of a CHW to be clearly delineated. and there is a system in place to identify and address situations where additional team support is needed.**

| Strongly disagree | Disagree | Neutral | Agree | Strongly agree |
| --- | --- | --- | --- | --- |

57. **It is possible for there to be a system in place to identify and address situations where additional team support is needed.**

| Strongly disagree | Disagree | Neutral | Agree | Strongly agree |
| --- | --- | --- | --- | --- |

**TRAINING AND SUPERVISION**

**Now, we are going to ask about your level of knowledge regarding the training and supervision of CHWs.**

**YES (conditional from Q11: IF YES TO QUESTION 11)**

58. **Our CHW(s) has undergone CHW certification training and/or state accreditation.**

- Yes
- No
- Not sure

59. **Our CHW(s) has completed the CDC Epilepsy and Self-Management Training for CHWs.**

- Yes
- No
- Not sure

60. **Our CHW(s) is supervised by (Check all that apply)**

- Hospital administrator
- Physician
- Epilepsy nurse specialist
- Social worker
- Community based organization
- Other
- Not sure

61. **Our CHW(s) has sufficient supervision and guidelines to ensure that they are providing the appropriate level of non-clinical care.**

| Strongly disagree | Disagree | Neutral | Agree | Strongly agree |
| --- | --- | --- | --- | --- |

62. **Our epilepsy center has an effective system of monitoring patient referrals to our CHW.**

| Strongly disagree | Disagree | Neutral | Agree | Strongly agree |
| --- | --- | --- | --- | --- |

63. **There is a standardized way to record and monitor which patient needs were adequately addressed by the CHW at our epilepsy center.**

| Strongly disagree | Disagree | Neutral | Agree | Strongly agree |
| --- | --- | --- | --- | --- |

64. **There is a protocol to collect feedback on patient experience with the CHW at our epilepsy center.**

| Strongly disagree | Disagree | Neutral | Agree | Strongly agree |
| --- | --- | --- | --- | --- |

**NO (conditional from Q11: IF NO TO QUESTION 11)**

65**. A CHW(s) hired at our epilepsy center must have CHW certification training and/or state accreditation.**

- Yes
- No
- Not sure

66. **A CHW(s) hired at our epilepsy center must complete the CDC Epilepsy and Self-Management Training for CHWs.**

| Strongly disagree | Disagree | Neutral | Agree | Strongly agree |
| --- | --- | --- | --- | --- |

67. **A CHW(s) hired at our epilepsy center would be best supervised by,**

- Hospital administrator
- Physician
- Nurse
- Social worker
- CHW leader at our medical center
- Other: ______________________________
- Not sure

68. **We would be able to provide sufficient supervision and guidelines to ensure that a CHW is providing the appropriate level of non-clinical care to epilepsy patients.**

| Strongly disagree | Disagree | Neutral | Agree | Strongly agree |
| --- | --- | --- | --- | --- |

69. **Our epilepsy center currently has an effective system that could be used to track patient referrals to a CHW and their ability to meet patient needs.**

| Strongly disagree | Disagree | Neutral | Agree | Strongly agree |
| --- | --- | --- | --- | --- |

**Funding to Support and Sustain a CHW Role on the Epilepsy Center Team**

**Please answer the following questions regarding your understanding of how the CHW position is funded and the sustainability of a CHW on the Epilepsy Center team.**

**YES (conditional from Q11: IF YES TO QUESTION 11)**

70. **Our CHW(s) is/are funded by (Select all that apply)**

- - Medical reimbursement
  - Granting mechanism (state, federal, other)
  - Municipal funds
  - Other ___________________
  - Not sure

71. **If medical reimbursement, by whom and what is covered?**

- Medicaid (full)
- Medicaid (limited) – specify what is covered: _____
- Medicaid contract with managed care organization
- Other

72. **There is a funding mechanism that supports sustainability of a CHW at our epilepsy center**

| Strongly disagree | Disagree | Neutral | Agree | Strongly agree |
| --- | --- | --- | --- | --- |

**NO (conditional from Q11: IF NO TO QUESTION 11)**

73. **It would be possible to fund a CHW at our epilepsy center through,**

- - Medical reimbursement
  - Department funds
  - Granting mechanisms (state, federal, other)
  - Municipal funds
  - Other _____________________
  - Not sure

74. **If medical reimbursement for services provided, by whom and what is covered?**

- Medicaid (full)
- Medicaid (limited) – specify what is covered: _____
- Medicaid contract with managed care organization
- Other

75. **A mechanism to support long term sustainability of a CHW role on our epilepsy center team is possible.**

Definitely not Unlikely Unsure Likely Very likely

**Epilepsy Team - Care Gaps**

**These questions will ask about your knowledge related to the Social Determinants of Health (SDOH) needs of your patients.**

76. **Please select from the following the perceived needs of your patients that require additional support currently not available through your epilepsy center,**

| Baby and family support services | Health education/Epilepsy Specific   - Medication adherence - Self-Management - Seizure Precautions/Safety - Seizure tracking - Pregnancy Planning - Comorbidities - Other |
| --- | --- |
| Behavioral health | Health insurance |
| Child care | Housing |
| Dental care | Interpersonal safety |
| Education | Legal |
| Employment / Unemployment filing for SSI, disability | Medical home |
| Financial assistance | Transportation |
| Food insecurity | Other |

77. **Currently,** **staff members who take on the role of filling gaps in care that relate to Social Determinants of Health (SDOH) at our epilepsy center include,**

- Nursing
- Social work
- CHW
- Administrative staff (PCAs)
- Trainees (residents, fellows)
- Other ________________

78. **Addressing SDOH through our epilepsy clinic is part of routine patient care.**

| Strongly disagree | Disagree | Neutral | Agree | Strongly agree |
| --- | --- | --- | --- | --- |

79. **Our resident and fellow trainees receive the appropriate education on SDOH in epilepsy.**

| Strongly disagree | Disagree | Neutral | Agree | Strongly agree |
| --- | --- | --- | --- | --- |

80. **My patients would benefit from a dedicated non-medical team member who can address SDOH.**

| Strongly disagree | Disagree | Neutral | Agree | Strongly agree |
| --- | --- | --- | --- | --- |

81. **SDOH needs that patients have should be addressed by their primary care provider office.**

| Strongly disagree | Disagree | Neutral | Agree | Strongly agree |
| --- | --- | --- | --- | --- |

82. **Addressing social issues in my patients’ life (such as housing, transportation, or employment) will improve my patients’ health.**

| Strongly disagree | Disagree | Neutral | Agree | Strongly agree |
| --- | --- | --- | --- | --- |

**Expectations and Trust**

**These questions will ask how confident you are in the ability of a CHW to help your patients.**

83. **If a CHW consultation was available at your epilepsy center, how likely is it that you would refer your patients to a CHW?**

| Extremely likely | Likely | Neutral | Not likely | Never |
| --- | --- | --- | --- | --- |

84. **I see the potential benefit of integrating a CHW onto our epilepsy team.**

| Strongly disagree | Disagree | Neutral | Agree | Strongly agree |
| --- | --- | --- | --- | --- |

85. **I would be confident in a non-medical person, like a CHW, assisting with patient support including handling sensitive health related, financial and other personal information.**

| Strongly disagree | Disagree | Neutral | Agree | Strongly agree |
| --- | --- | --- | --- | --- |

86. **If a CHW were trained in epilepsy specifically, my confidence level for their ability to help would be,**

- Very high
- High
- Moderate
- Low
- Very low

87. **It is possible for a member of our epilepsy team to effectively supervise a CHW**

| Strongly disagree | Disagree | Neutral | Agree | Strongly agree |
| --- | --- | --- | --- | --- |

88.  **The epilepsy patients I care for would welcome support from a CHW.**

| Strongly disagree | Disagree | Neutral | Agree | Strongly agree |
| --- | --- | --- | --- | --- |

89. **I have confidence a CHW would act in the best interest of the patients I provide epilepsy care to.**

| Strongly disagree | Disagree | Neutral | Agree | Strongly agree |
| --- | --- | --- | --- | --- |

**Center environment, collaborative culture**

**We would like you to think about the opportunity to have a CHW as part of your epilepsy center. We will ask you what you think about working with a CHW.**

90. **In order to be incorporated and accepted into the healthcare team, a CHW should be based in the clinic at the epilepsy center.**

| Strongly disagree | Disagree | Neutral | Agree | Strongly agree |
| --- | --- | --- | --- | --- |

91. **CHWs should attend epilepsy center team group meetings (i.e. nursing rounds reporting).**

| Strongly disagree | Disagree | Neutral | Agree | Strongly agree |
| --- | --- | --- | --- | --- |

92. **CHWs should maintain a connection with CHW training organizations and meet with other local CHWs for ongoing support and guidance.**

| Strongly disagree | Disagree | Neutral | Agree | Strongly agree |
| --- | --- | --- | --- | --- |

93. **CHWs should provide feedback to the clinical epilepsy team concerning clinical care gaps.**

| Strongly disagree | Disagree | Neutral | Agree | Strongly agree |
| --- | --- | --- | --- | --- |

94. **CHWs should provide information to the clinical team on high-risk patient populations in the epilepsy clinic including those that are socially and/or economically disadvantaged.**

| Strongly disagree | Disagree | Neutral | Agree | Strongly agree |
| --- | --- | --- | --- | --- |

95. **CHWs can play a role in educating the members of an epilepsy center team around community-based supports and resources available to people with epilepsy.**

| Strongly disagree | Disagree | Neutral | Agree | Strongly agree |
| --- | --- | --- | --- | --- |

96. **If trained to do so, CHWs are capable of delivering a standardized epilepsy self-management program to patients.**

| Strongly disagree | Disagree | Neutral | Agree | Strongly agree |
| --- | --- | --- | --- | --- |

97. **A mechanism (electronic medical record integration, weekly rounds) for CHWs to provide direct feedback to clinical providers on the epilepsy center team about patients whose care they participate in is necessary to achieve the highest level of patient care.**

| Strongly disagree | Disagree | Neutral | Agree | Strongly agree |
| --- | --- | --- | --- | --- |

98. **A CHW can help my patients to achieve better health and well-being.**

| Strongly disagree | Disagree | Neutral | Agree | Strongly agree |
| --- | --- | --- | --- | --- |
